# Exploring the impact of COVID-19 on substance use patterns and service access of street involved individuals in Kingston, Ontario: a qualitative study

**DOI:** 10.1186/s12889-022-12976-6

**Published:** 2022-03-23

**Authors:** Victoria McCann, Rachael Allen, Eva Purkey

**Affiliations:** 1 Queen’s University School of Medicine, Kingston, ON K7L 3N6 Canada; 2 Department of Family Medicine, Queen’s University, Kingston, ON K7L3G2 Canada

**Keywords:** Substance use, COVID-19, Service access, Street involved, Opioid epidemic, Harm reduction, Qualitative research, Public health

## Abstract

This study aims to understand the experiences of street-involved individuals during the COVID-19 pandemic regarding substance use patterns and service access. With the collision of the COVID-19 pandemic and Canadian opioid epidemic came an increase in opioid related overdoses and increased barriers in accessing essential services since March 2020. Semi-structured interviews were conducted in June and July 2021, with 30 street-involved individuals in Kingston, Ontario. Analysis followed a phenomenological approach to qualitative research. Themes were coded by two independent researchers using NVIVO12. COVID-19 had detrimental effects on the lives of street-involved folks who use substances. Increased substance use to combat feelings of isolation and hopelessness related to loss of income and housing was commonly described. Increased fentanyl usage was considered the major contributor to the rise in overdoses over the pandemic. Restrictions on public access to businesses and services disproportionately impacted individuals with limited means. Harm reduction services and mental health support were considered extremely important throughout the pandemic. The coinciding COVID-19 pandemic and opioid epidemic place street-involved individuals who use substances in a uniquely dangerous position. As such, it is imperative that public policy decision-makers consider the differential needs of street-involved community members to provide safe, relevant, and compassionate solutions in future public health emergencies.

**Supplementary Information:**

The online version contains supplementary material available at 10.1186/s12889-022-12976-6.

## Background

It is estimated that 21% of Canadians will meet the criteria for substance use disorder at some point in their lifetime [1]. In Ontario, roughly 10% of people are either dependent on substances or use substances in a way that interferes with their daily life [2]. Due to the ongoing criminalization and stigmatization of drug use, people who use substances often experience marginalization and a disproportionate number experience poverty, homelessness, and incarceration [3]. Canada has weathered an ongoing opioid epidemic claiming over 21,000 lives between 2016 and 2020 [4]. This pre-existing epidemic has been exacerbated by the coinciding COVID-19 pandemic declared on March 11th, 2020 [5]. Between April 2020 and March of 2021, Canada saw an 88% increase in apparent opioid toxicity deaths compared to the same time frame in 2019–2020 [4]. Among people experiencing homelessness, Ontario saw the proportion of opioid deaths rise significantly from 11.6% to 16.0% between March 2020 and December 2020 compared with the year prior [6].

Individuals with substance use issues have faced immediate challenges in the context of the coronavirus pandemic. Among government efforts to slow the spread of COVID-19 were widespread limitations to accessing public services and community supports [5, 7]. Reduced capacity and longer wait times at specialized substance use treatment centres in the early months of the pandemic posed significant barriers to individuals accessing care such as counselling or group therapy and contributed to an increase in relapses [7, 8] Accessing basic needs, harm reduction, and behavioural health services became more difficult due to changes in operating hours, limits to occupancy, and restrictions in public transportation [7, 8]. People who use substances also experience higher rates of mental health issues [9] which were compounded by heightened feelings of isolation and hopelessness as well as restrictions on reaching mental health services and personal support workers during the COVID-19 pandemic [10]. Some healthcare and social services transitioned their appointments to virtual models in order to adhere to physical distancing, but this created barriers for individuals who do not have regular access to a phone or electronic device [11]. Additionally, lockdown measures altered the social context in which people were able to use substances with more individuals finding themselves socially isolated and consequently using substances alone [6, 7]. Limited access to a drug of choice in a certain area may lead to individuals opting to use different substances or different suppliers that they are less familiar with, therefore increasing their risk of overdose [8]. Adulteration and increased toxicity of substances has been observed across Canada [4, 5]. Ontario reported an increased proportion of accidental opioid overdoses in a peri-pandemic cohort (March 2020 – December 2020) compared to a pre-pandemic cohort (March 2019 – December 2019) of which fentanyl was the primary substance and accidental overdose was the primary mode [6]. While this data supports the understanding that the overdose epidemic has worsened since the introduction of widespread community restrictions, a qualitative lens will contribute to the understanding of the changes in substance use patterns and access to vital community services on the level of the individual.

Kingston, a community of 136,000 people, is located within 300 km of three major cities in Southeastern Ontario; Toronto, Ottawa, and Montreal. It has weathered some of the strictest lockdown measures in the world since the onset of the pandemic while simultaneously experiencing an extremely low case count and mortality rate from COVID-19 relative to the rest of Ontario and Canada [12]. The Integrated Care Hub (ICH) opened in Kingston approximately 6 months into the pandemic, as a response to the increased needs of street involved people in the area and in reaction to homeless encampments that developed over the summer of 2020 [13, 14]. The term ‘street involved’ in the context of this population refers to individuals who may experience varying degrees of homelessness at any given time as well as a range of at-risk behaviours. The ICH functions as a supervised consumption site, shelter, food kitchen, drop-in space and point of communication between clients and additional services in the community. Despite efforts made to ensure basic needs were met, access to vital community resources and services changed in accordance with provincial public health guidelines since the onset of the pandemic in March 2020. The narrative experiences of street involved people who use substances during the collision of the COVID-19 and opioid crises are absent from the current discourse and need to be investigated. Through analysis of the narrative voices of street involved community members who access the services at the ICH in Kingston, Ontario, this study aims to illuminate the disruptions in access to services and the changing patterns of substance use since the onset of the COVID-19 pandemic. Findings from this study will contribute to the body of knowledge related to the joint impacts of the COVID-19 pandemic and the opioid epidemic, and will also serve to inform policy-makers regarding the unique needs and challenges faced by street involved populations in the context of potential future lockdowns and community restrictions.

## Method

### Study Design

Phenomenological studies combine the lived experiences of key informants to define the essence of a specific phenomenon [15]. In this study, a phenomenological qualitative approach was used to explore how the COVID-19 pandemic has impacted the substance use patterns of street involved persons in Kingston, Ontario. Semi-structured interviews were conducted using questions focused on participants’ experience with substance use before versus during the pandemic, as well as their ability to access services and basic needs throughout the pandemic. The interview guide was created under the guidance of a family physician and researcher, (EP), who has experience working with street involved community members, as well as staff at the Integrated Care Hub who work with this group on a daily basis. This study received Queen’s University Health Sciences & Affiliated Teaching Hospitals Research Ethics Board approval and was completed in collaboration with Kingston’s Integrated Care Hub.

### Participant Recruitment

Participants were recruited from the ICH. As mentioned above, the ICH is a safe consumption site, a 25-bed shelter, and a drop-in space, accessible 23 h a day, offering food and mental health services among others. Prospective participants were approached non-selectively on the property outside the ICH and offered participation in the study. Researchers introduced themselves, provided details on the purpose of the study, explained the protocol for maintaining privacy, and obtained informed consent. Individuals were excluded if they were a) under 18 years old, b) in visible distress or decompensation, or c) unwilling to don a face covering in the context of COVID-19 research protocol precautions. Two researchers (VM and RA) conducted semi-structured interviews using the semi-structured interview guide (Supplementary Material). Basic demographic data was also collected during the interviews, including age, gender, current sources of income, and immediate plans for shelter (Table 1). At the conclusion of the interview, participants were compensated with $25 cash to thank them for their time and contributions.

**Table 1:** Demographics

Variable	Response, n = 30 (%)
Age	
< 21	1 (3.3)
21 to 29	7 (23.3)
30 to 39	7 (23.3)
40 to 49	4 (13.3)
50 to 59	7 (23.3)
60 to 69	4 (13.3)
70 +	0
How do you describe your gender identity?^a^	
Male	23 (76.7)
Female	7 (23.3)
What are your current sources of income?	
Employed	
Part time	1 (3.3)
Self-employed	1 (3.3)
Odd jobs	1 (3.3)
Ontario Disability Support Program (disability pension)	17 (56.7)
Panhandling	9 (30.0)
Ontario Works (welfare)	8 (26.7)
Selling drugs	8 (26.7)
Canada Pension Plan	2 (6.7)
Investment income	1 (3.3)
Sex work	1 (3.3)
Employment insurance	1 (3.3)
Binning (collecting cans from garbage pails or recycling bins)	1 (3.3)
Where do you plan to sleep tonight?	
Shelter (incl. Integrated Care Hub)	23 (76.7)
Own home/ apartment	4 (13.3)
Outside	1 (3.3)
Friend’s place	1 (3.3)
Won’t be sleeping	1 (3.3)

^a^Open-ended question

Participants could indicate more than one response therefore totals may not add up to 100%

### Data Collection and Analysis

Data collection was completed in June and July 2021. Interviews were audio recorded and transcribed verbatim with anonymization and audio recordings were deleted following transcription. Transcripts were imported into NVIVO12 [16] and coded line-by-line independently by two researchers (VM and RA). As this is an exploratory study, open coding thematic analysis was performed to allow for the emergence of meaningful themes from the data itself. Initial codes were defined by thoroughly reading the transcripts and identifying key descriptions. Researchers then grouped multiple codes to derive the key themes. Codes and themes were compared between researchers to ensure consistency.

## Results

A total of 30 participants were interviewed on the property of the ICH. The duration of the interviews ranged from 14 to 63 min with the average being 32 min in length. The sample population was majority male, (23 M, 7F), with an age range of 21 to 69 years. Participants’ personal sources of income were the Ontario Disability Support Program (disability pension) (56.7%), panhandling (30.0%), Ontario Works (welfare) (26.7%), and drug selling (26.7%). Majority of participants were using the ICH as a shelter (76.7%). Other participants had plans to sleep at their own apartments (13.3%), outdoors (3.3%), or at friends' residences (3.3%). The most commonly reported substances being used were marijuana (80.0%), crystal meth (76.7%), and fentanyl (53.3%). Complete demographic and survey findings are presented in Tables 1, 2, and 3.

**Table 2:** Service Utilization and Substance Use

Variable	Response, *n* = 30 (%)
Services used since the start of COVID-19	
Shelters	26 (86.7)
Drop-in spaces	26 (86.7)
Hospitals	23 (76.7)
Food banks	21 (70.0)
Soup kitchens	20 (66.6)
Harm reduction services	19 (63.3)
Emergency departments	19 (63.3)
Housing supports	17 (56.7)
Health clinics	13 (43.3)
Services to help obtain ID	13 (43.3)
Legal services	9 (30.0)
Job training	6 (20.0)
Detox	6 (20.0)
Rehab	1 (3.3)
Other	
Dentist	1 (3.3)
Elder	1 (3.3)
Mental health services	1 (3.3)
Methadone clinic	1 (3.3)
Personal nurse	1 (3.3)

Participants could indicate more than one response therefore totals may not add up to 100%

**Table 3:** Substance Use

**Variable**	**Response,** n = **30**
Substances used since the start of COVID-19	
Marijuana	24 (80.0)
Crystal meth	23 (76.7)
Fentanyl	16 (53.3)
Alcohol	16 (53.3)
Hydromorphone	14 (46.7)
Morphine	13 (43.3)
Benzodiazepines	12 (60.0)
Cocaine	11 (36.7)
Methadone/ suboxone	10 (33.3)
Crack	9 (30.0)
Amphetamines/ stimulants	8 (26.7)
Heroin	7 (23.3)

Participants could indicate more than one response therefore totals may not add up to 100%

### Thematic Analysis

While this research was intended to focus on the changes in substance use patterns of street involved persons consequent to the COVID-19 pandemic, the semi-structured nature of the interviews allowed for the emergence of themes auxiliary to the initial research focus. The interview questions were phrased such that participants were asked to compare present behaviours and perceptions to pre-pandemic. After thematic analysis, five themes were identified.

### Theme 1: Changes in patterns of substance use with the COVID-19 pandemic

Most participants described an increase in the overall use of substances as a result of the pandemic, especially an increase in the use of fentanyl. While a small minority reported a personal decrease in substance use since the start of the pandemic, the overall consensus among participants was an observed increase in substance use within the community.

“[People are using substances] more because they ain’t got nothing to fucking do, everything is closed. Man, there are so many people here who I didn’t think… I just thought they were homeless… But these are people who actually had jobs and careers and shit like that and lost their jobs… And you didn’t expect it. I can see four or five people over there right now behind you that had good paying jobs, decent fucking… before the pandemic hit and now they’re here in the shelter. Fighting for beds.”

Participant 108; male, 38 years old

Interviewees experienced job loss, the loss of typical responsibilities, and the disintegration of purposeful daily routines. Lockdowns and the overall climate of the COVID-19 pandemic were reported to be associated with feelings of helplessness, hopelessness, depression, and stress. To combat these feelings, many participants described seeking greater overall substance use, including the numbing effect of fentanyl. Relatedly, accidental overdoses and deaths linked to fentanyl were reported as having greatly increased over the course of the pandemic. Of note, while the subject of accidental overdoses and fentanyl related were discussed in depth, there was little mention overall of participants’ fears of catching the COVID-19 virus themselves.

“People are more helpless so [substances] are more dangerous. And the places where you get help are closed down. You can’t get as much help. It’s more dangerous with COVID, yeah.”

Participant 922; male, 56 years old

“[People are doing fentanyl alone because] they’re just depressed. Sick of living. [This year is] depressing everybody. Especially with the mental health.”

Participant 289; male, 23 years old

“…Money was going towards more, like, bringing more fentanyl into the city you know what I mean? And it’s killing more people, more people are dying and it’s not just from COVID, they’re dying from the fentanyl use. Probably 80, no... 60 percent of the people here are on fentanyl, you know what I mean? And that’s sad to say. It used to be a crystal meth town and now it’s pure fentanyl, you know? ... And I started using fentanyl and I don’t know why, but I dunno, it’s the easiest thing to get.”

Participant 872; female, 32 years old

### Theme 2: The relative meaning of “essential” and the impact of COVID-19 restrictions on those with limited means

Many individuals expressed strong frustrations with certain COVID-19 restrictions as they differentially affected lower-income and unhoused persons. Public health efforts to curb the spread of COVID-19 included the closure of public indoor spaces, such as restaurants, libraries, and some retail stores. In retail stores that remained open for purchasing essential items, such as food and hygiene products, accessing items that were deemed non-essential was restricted (i.e. certain stores had aisles that were blocked off in which “non-essential” products could not be accessed).

“How they closed non-essential…like some of us have gone without socks for weeks and we needed socks. Could have bought them at the dollar store or [name of Department store] but since it’s non-essential, you can’t. But yet, scissors and pencil crayons and stuff were essential. I find that just dumb.”

Participant 303; male, 26 years old

Participants described how many of the services and locations that were deemed unessential by public health were in fact essential for this population. Closures affected participants’ access to washrooms and places to practice personal hygiene. Affordable supplies, such as camping supplies and socks, were limited by retail store closures and restrictions on non-essential item purchasing. In particular, participants who identified as unhoused described these purchasing restrictions as severely impacting their feelings of safety and their well-being.

“People need clothes. People do need to get new clothes sometimes, you know? Little things, like it’s hard to explain. Like when we come here,[...], a lot of people’s shit gets stolen so they have nothing. So where do they start again? There’s nothing open to start again.”

Participant 872; female, 32 years old

In addition, without reliable access to a mobile device or the internet, participants faced disproportionate barriers to accessing social and government-services. Legal aid clinics and welfare workers were unable to operate in-person due to COVID-19 restrictions, and court hearings were held on video conferencing platforms. The vast majority of participants did not own a cell phone and described how much of a challenge this was since the start of the pandemic.

“People have a stigma about homeless people and people without jobs or people in our situation that they don’t have nothing to do all day, you know what I mean, because they’re doing drugs they’re not doing anything. That’s the furthest thing from the truth. I wake up every day and I think how am I going to survive that day. Not just like, how am I going to pay for my vacation. I’m surviving, right. Every day is survival. I got to worry about eating today, I got to worry about a place to sleep. That’s my first priority. I’ve got tape on my fucking shoes right now because my feet are falling out of em. I walk everywhere, I can’t afford a bus, the bus pass doesn’t come to me, I don’t have [welfare] or [disability pension] or [student loans]… And everything is worked on a cell phone, and if I could keep a cell phone longer than an hour that would be cool.”

Participant 179; male, 37 years old

“It’s very difficult to access [services] if you don’t have a good phone. Like I don’t use my phone. I don’t have any extra money to put in my phone, well I do have extra money to do it, but I don’t like the idea of taking $25 and putting it on my phone.”

Participant 931; male, 61 years old

### Theme 3: The impact of COVID-19 restrictions on community support and services

Access to food, clothing and shelter were seen as the most important services in a pandemic. While needs for food were usually met, needs for consistent access to shelter were not. Indoor capacity limits at the ICH and other shelters were identified as a stressor for participants seeking the use of the shelter.

“Well they’ve cut down a lot on how many people are allowed to be in a room and stuff. ‘Cause half the people – ‘cause there’s more room but they’re not letting people in, they have to stay outside. That’s the part that really sucks.”

Participant 822; male, 50 years old

Participants detailed how their usage of the ICH impacted their ability to access and interact with family support, as families were concerned that, by using the ICH, individuals were at a greater risk of being exposed to COVID-19 because they were consistently around other people.

“[My mom] wants me to stay inside and not hang around other people. But the amount of people that are here [the Integrated Care Hub] is a lot, you know. Everybody is always around everybody. “

Participant 872; female, 32 years old

Extensive closures of in-person social services, the promotion of physical distancing, and limiting contacts resulted in an overall reduction of community and personal support felt by this study population. Participants further emphasized how the restrictions on in-person contact between themselves and their community workers, such as at-risk nurses, housing workers, and financial assistance managers, were detrimental to their well-being.

Participant: “[My relationship with my at-risk nurse] is really important for my well-being.”

Interviewer: “And when the nurse wasn’t able to take you out for coffee, how did that… How was that for you.”

Participant: “It hurt me, made me feel frustrated. Isolated. I wasn’t getting the care that I needed. He was doing the best he can but he’s got his rules.“

Participant 922; male, 56 years old

### Theme 4: Emerging supports and the importance of harm reduction during a pandemic

While the in-person operation of many services was being limited, the ICH was opened in November 2020 in response to the convergence of the opioid epidemic and COVID-19 pandemic. Having access to safety, shelter, food, and clothing during the pandemic were rated as highly important by participants. The impact of the safe consumption site was mentioned by most participants, as the ICH has provided life-saving support to over 400 overdoses since its opening [17].

“Right now, the only services I’ve noticed people using – which is a good thing too – is the safe injection site, the CTS [Consumption and Treatment Services], they’re amazing. [...] anybody that has come to the CTS to use during the COVID...their lives have been saved. Every single time. Not one person has died in these people’s hands.”

Participant 795; female, 39 years old

Participants also described how community members have generally migrated from consuming substances out in the community to using at the safe consumption site. Overall, the opening of the ICH was discussed positively by nearly every person interviewed.

“They do have good resources here [at the Integrated Care Hub]. I got a mental health worker here. And I got in touch with the hepatitis clinic here. And I got on methadone here. I’ve been here myself for 12 days now, and 12 days I accomplished a few good things anyways. I got a mental health counselor, a normal counselor, an abusive counselor, a civil lawyer for my accident, and hepatitis clinic, a hepatitis counselor, and got on methadone since I’ve been here. And they’re going to try to help me get onto housing so I can get a place.”

Participant 872; female, 32 years old

“Yeah for sure people are using the hub [The Integrated Care Hub], everyone’s using the hub. Everyone’s coming to use here. It’s good and it’s saving lives, it’s a good thing. A lot of people died over the past year and they shouldn’t have died. A lot of people have died in the dirty ways too, but yeah. Been killed because fentanyl is such an easy… such a killer drug.”

Participant 108; male, 38 years old

“Well I think [substance use] is a little less dangerous in a way because everyone’s not going out and about and getting dope elsewhere, they’re coming here to the CTS [Consumption and Treatment Services] so if it keeps them coming to the CTS then at least they can make sure that they’re okay, they’re okay, they go on and do it safely, and that part of it, they make sure they survive. You know because doing, you know, fentanyl or heroin, could end your life.”

Participant 952; male, 25 years old

### Theme 5: Complex interpersonal affairs between clients

Despite the positive impacts of the ICH mentioned above, participants described the ICH and surrounding premises as an environment where the consumption of substances is ubiquitous and often associated with conflict between patrons. Substances are used openly, drug paraphernalia is left about, and participants recovering from substances described the constant encounter with them as challenging.

“I really hate it here. It’s really bad here. Like drugs, drugs, drugs. For the time you wake up until the time you go to sleep. That’s all people talk about. Like “Can I get a toke,” “Do you know where to get anything,” “Can I get anything?” That’s all the questions. And even if you’re by yourself and you want to be left alone. The more you’re by yourself, the more you’re left alone the more people come up to you. And bother you. You know what I mean? And they think that you’re just keeping your dope for yourself. And really you’re just not – you don’t have nothing and you’re just trying to stay by yourself you know?”

Participant 872; female, 32 years old

“Hotshotting”, the tainting of injectable drugs with insulin to intentionally cause harm to another person, was highlighted as a common issue in interpersonal conflicts. Theft of personal belongings, either while accessing the shelter or during periods of opioid-induced drowsiness, was also an issue experienced by participants. Participants explained that, by having all of the wrap-around services in one place, continuous use of the ICH was related to complex interpersonal conflict.

“Well when you have a shelter the worst thing to do, especially being homeless and on the street, is have an injection unit underneath. But it’s also safer.”

Participant 610; female, 39 years old

On the other hand, participants also described the importance of community within this population. Narratives of helping to save the lives of fellow patrons, administering naloxone and CPR, and the value of relationships formed with staff and other patrons of the ICH were highlighted. Some voiced desires for more opportunities to come together as a community in a positive way in order to find purpose and joy in the form of social events and fundraisers.

“I’ve known a lot of these people for years. It’s all the same group of people, misfits of the community. They’re not really bad people, they are definitely not. I know people who are great people, I’ve saved a lot of these people's lives and there’s a reason why. Not just because I just like them, but because they deserve it. Everybody deserves life. The sense of community here, it’s one of those things that you don’t really obtain in just any community.”

Participant 179; male, 37 years old

 “I believe in the power of healing and my energies and stuff like that. And so for my mental health I nurture it the best that I can myself. And ... for like my friends, a lot of people that I care about–I care about everybody, period. But lacking like – I think they should have more events. More community events where people can come together. And not feel so individualized. I think coming to you know your regular appointments, see your counselor or whatever it may be, I think honestly people would really benefit – and I’m saying this because I love a lot of people, I really do here, and to see all of this is... it’s taking a toll on all of us.”

Participant 795; female, 39 years old

## Discussion

Our findings present the experiences of a subset of street involved individuals since the start of the COVID-19 pandemic with respect to substance use and service utilization. These findings enhance existing quantitative research and provide insight into the experiences and struggles of people who use substances in a time of relative resource deprivation and public health emergency. The heightened vulnerability to contracting the virus [18] and detrimental effects of COVID-19 restrictions on people who are street involved and use substances led many to fear the collision of the COVID-19 pandemic with Canada’s ongoing opioid epidemic [19, 20]. Our findings are consistent with and support a rapid needs assessment report carried out by the Integrated Care Hub between February and April 2021 [21].

Kingston, Ontario, experienced some of the lowest COVID-19 case rates across Canada [22] while still enduring some of the strictest provincial guidelines in terms of lockdowns and service restrictions [12]. Meanwhile, the city continued to battle the ongoing opioid epidemic at an escalating rate [21]. Between March 16 and December 31, 2020, Kingston had 0 deaths from COVID-19 and 33 deaths from opioid overdoses––an increase from 22 opioid-related deaths in the same period in 2019 [6, 22]. Among participants, there was relatively little mention of fears of becoming sick from the COVID-19 virus, however, opioid overdoses had touched the lives of every single person interviewed at the ICH. Additionally, it was clear how many people wanted to have their voices heard. At times, participants waited as long as 3 h to be interviewed, and other times people had to be turned away because of time constraints for the day.

The consensus among study participants was that substance use overall had increased since the beginning of the pandemic and fentanyl use had increased in particular. This supports data from a Public Health Ontario report on opioid toxicity which describes fentanyl as the primarily isolated substance in 87.0% of deaths between March 16th and December 31st of 2020, a 12.0% increase compared to the pre-pandemic cohort March 16th and December 31st, 2019 [6]. The growing prevalence of fentanyl was viewed with negativity, frustration, and sadness, even among participants who consistently used fentanyl themselves. Accompanying this was the observation that increased fentanyl usage paralleled increased feelings of hopelessness and isolation. Underlying many of the mental health struggles discussed were themes of job loss, housing loss, and loss of purpose that came with the pandemic. While these feelings are not exclusively experienced by street involved people who use substances, according to our findings and others, these circumstances come with the heightened dangers of increased fentanyl use as a means to numb or forget the painful experiences of everyday life [21, 23]. This accentuates the need for mental health resources, affordable housing, and meaningful employment opportunities as a top priority as support during both the COVID-19 pandemic and the ongoing opioid epidemic.

Housing continues to be a major struggle among street involved community members in the face of the pandemic in Kingston and elsewhere [7, 21, 23]. Our participants repeatedly described how finding affordable, adequate, and accessible housing became harder due to restrictions in capacity and loss of income. The relative percentage of those experiencing homeless at the time of opioid-related overdose death in Ontario increased significantly from 11.6% to 16.0% between pre- and post-pandemic cohorts [6].

Province-wide lockdowns and public health protocols to prevent the spread of COVID-19 virus profoundly changed the daily lives of street involved people in Kingston. While these protocols proved to be highly effective at reducing spread of the virus, individuals with limited means were differentially affected than members of the general population. As many health and legal appointments transitioned to telehealth and virtual platforms, people without access to technology were either forced to forgo these appointments or make use of communal devices, often resulting in inefficient and delayed communication. This highlights the continuous need for flexible, in-person, and low-barrier access to appointments until technology is universally accessible as a civic right [7, 23]. Other restrictions that were put in place to avoid non-essential shopping and social gathering resulted in items such as clothing and camping supplies being deemed non-essential, and facilities such as libraries, community centres, and public restrooms being closed to public access. These policies overlooked the needs of precariously housed individuals who do not have consistent access to clothing, shelter, and hygiene facilities. Overall, participants in this study did not feel that the City of Kingston understood their needs, or which services were “essential” to them, further broadening the divide between policy makers and service end-users. The discrimination denounced by interviewees is not only a risk factor for immediate psychological and personal harm, but is also associated with dysregulation of immune function and elevation of inflammatory markers, predisposing this population to negative long-term health consequences [24]. This supports the importance of minimizing the discrimination against this population in public health policy in the future.

One major triumph for Kingston’s harm reduction advocates was the emergence of the multifunctional Integrated Care Hub (ICH) eight months into the pandemic. The ICH meets a vast range of community needs and underscores the importance of a central, integrated, and judgement-free source for services and supports aimed at people experiencing homelessness, food insecurity, and substance use. While many communities across Canada have seen a significant rise in opioid-related deaths since the start of the pandemic [25], the opening of the ICH in late 2020 demonstrably mitigated the same rise in deaths from occurring in Kingston [6, 21]. In January of 2021, the ICH reversed more overdoses than 2018 and 2019 combined [21]. The importance and impact of harm reduction services in Kingston were discussed by the majority of participants, in particular the success with which the ICH has reversed opioid overdoses. These findings support accessible harm reduction services as vitally essential services amidst a public health emergency.

### Future Directions

This area of research would benefit from hearing from members of the community who are street involved but do not access the community support at the ICH. Without immediate access to the safe consumption site, drug testing, clean needles, naloxone, and on-site paramedics, these individuals may be at an increased risk for negative outcomes from substance use, or may have developed alternate protective mechanisms. A better understanding of their experiences would help address further barriers to accessing safe care and supports.

### Strengths and Limitations

A strength of this study is that it centres the narrative voices of those with lived experiences of substance use and street involvement. The opportunity to share personal experiences and amplify these voices to improve the current body of knowledge not only serves as a way to explore nuanced ideas but also build trust and relationships where they did not exist before. The limitations of this study include the fact that substance use is a highly sensitive and stigmatized topic. It is therefore possible that participants might not be completely forthcoming with their experiences and responses may be subject to social desirability bias. There was the potential for selection bias based on our inclusion criteria, as we could not include individuals exhibiting signs of acute distress or those unable/unwilling to wear a mask for the interview as a safety precaution for our interview team. There is also inherent subjectivity in the analysis of qualitative research. This was minimized by independent coding by two separate authors, however, cannot be eliminated completely. Additionally, we asked participants to recall their experiences over a long time frame (March 2020—June/July 2021) and many focused on more recent experiences versus experiences that may have happened earlier in the pandemic. Lastly, the generalizability of this study is limited by the uniqueness of the population at the Integrated Care Hub and the setting of Kingston, Ontario. Kingston is a medium-sized, urban centre that experienced few COVID-19 cases relative to the provincial averages but underwent the same restrictions as all of Ontario. The interviews took place after many public health measures had been lifted and vaccines had become available, between the third and fourth waves of the pandemic, and therefore may have impacted perceptions regarding the impacts of COVID-19 and evolving access to services.

## Conclusions

This study presents several messages with respect to the experiences since the start of the pandemic of street involved individuals who use substances. First, this population wants to have their voices contribute to the conversation regarding the needs of their community in the context of a public health emergency. Second, addressing the needs of this community in the form of mental health resources, affordable housing, and meaningful employment opportunities should be considered a top priority in mitigating the trends of increased substance use since the onset of the pandemic. The housing crisis in particular requires the attention of policy-makers to ensure safe solutions are in place to prevent isolation and high-risk substance use. Third, it is important that harm reduction services be readily accessible at all times, especially during times of public health emergencies. Lastly, as more services shift towards online platforms, there remains a continuous need for low-barrier, flexible and in-person services and appointments to consider the needs of individuals who do not have consistent access to phones or the internet. The differential needs and “essential services” of individuals who are street involved and use substances have not been adequately considered up until this point in the global pandemic and warrant considerable attention to improve the lives of and relationships with this community.

## Supplementary Information

**Additional file 1. **

## Data Availability

The datasets analyzed during the current study are not publicly available to protect the privacy of participants but are available from the corresponding author on reasonable request.
