# Supplementary material for: Exploring the impact of COVID-19 on substance use patterns and service access of street involved individuals in Kingston, Ontario: a qualitative study
Source: BMC Public Health. 2022 Mar 23;22:571. doi: 10.1186/s12889-022-12976-6 (PMC8940590; doi:10.1186/s12889-022-12976-6)
Supplement: Supplementary file 1 — Additional file 1. [file 12889_2022_12976_MOESM1_ESM.docx]

**Semi-Structured Interview Guide**

*Screening questions*

1. How old are you?
2. Have you spent more than 3 months away from Kingston for the past 12 months?
3. In the last 12 months, have you, or someone you know well used substances?

*Demographic information*

1. How would you describe your gender identity?

1. What sources of income to you have?
   1. Full-time employment
   2. Part-time employment
   3. Seasonal employment
   4. Informal employment (i.e. side jobs here and there)
   5. ODSP
   6. Panhandling
   7. Sex-work
   8. Drug Dealing
   9. Investment income
   10. Ontario works
   11. Employment insurance
   12. Canada pension plan
   13. Guaranteed income supplement
   14. Old-age security
   15. None
   16. Other
2. Where will you be sleeping tonight?
   1. Own home/apartment
   2. Family/friend’s home
   3. Couch surfing
   4. Shelter
   5. Vehicle
   6. Outside (sleeping rough)
   7. I don’t know
3. What neighbourhood of Kingston do you most often stay in?

*Interview topics*

1. Have you, or the person you know, used any of the following services in the past 12 months?
   1. Detox
   2. Rehab
   3. Drop- in spaces
   4. Food banks
   5. Harm reduction services
   6. Health clinics
   7. Emergency departments
   8. Hospitals
   9. Housing supports
   10. Job training/job supports
   11. Legal clinic
   12. Services to help you get ID
   13. Shelters
   14. Soup kitchens
   15. Other

| Question/Topic | | Prompts |
| --- | --- | --- |
| Topic 1: Access to community services | | |
| Have you accessed community services since the start of the pandemic? | - Which ones? - Have you been able to consistently access them whenever you wanted to? | |
| In your opinion, what community services are the most important during a pandemic? | - Have any services been missing? - Are there any services you started using since the start of the pandemic that you didn’t use before? | |
| How did the community programs you accessed communicate changes with you? | - When the locations of services changed? - When types of services changed? - When services were shut down? - When in-person appointments became possible? | |
| Topic 2: Community Support | | |
| Did you have more or less trouble accessing any other essential services (i.e. food, housing) during the pandemic? |  | |
| Throughout this pandemic, do you feel like your needs have been considered by the City of Kingston? | - Do you feel like your needs have been considered by service providers? Healthcare providers? Public health? - How could these agencies do better? | |

1. Have you or someone you know used any of the following substances in the last 3 months?
2. Alcohol
3. Benzodiazepines
4. Cocaine
5. Crystal meth
6. Amphetamine/stimulants (eg., Adderall, Vyvanse)
7. Heroin
8. Morphine
9. Hydromorphone
10. Fentanyl
11. Marijuana
12. Methadone/Suboxone
13. Crack
14. Other

| Question/Topic | Prompts |
| --- | --- |
| Topic 3: Experience with substance use throughout the pandemic | |
| Can you describe a routine incident of substance use during the pandemic? | - Is this experience different during times of lockdown? |
| Can you tell me about the most memorable experience with substance use since the start of the pandemic? |  |
| Do you think using substances is more or less dangerous since the start of the pandemic? | - Why? What makes it more/less dangerous? |
| From your perspective, how has substance use in our community changed over the last year? | - Are people using substances *more or less* than they were before the start of the pandemic? - Have the *types of substances* that people are using changed during the pandemic?   - Which ones? How has this changed? - Have the *setting or location* of substance use changed during the pandemic? - Are people *using substances differently* than they were before? (i.e. smoking vs ingested vs injected) |
| What good or bad has come from the pandemic with respect to people using substances? |  |
| Can you tell us anything about overdoses that have happened since the start of the pandemic? |  |
| Closing & Final Questions:  *Thank you for taking the time to speak with us today.* | |
| Is there anything we missed? |  |
| What are the most important messages that we need to know as health and social service providers? |  |
